# Targeting the Neddylation Pathway to Suppress the Growth of Prostate Cancer Cells: Therapeutic Implication for the Men's Cancer

**DOI:** 10.1155/2014/974309

**Published:** 2014-06-29

**Authors:** Xiaofang Wang, Lihui Li, Yupei Liang, Chunjie Li, Hu Zhao, Dingwei Ye, Menghong Sun, Lak Shin Jeong, Yan Feng, Shen Fu, Lijun Jia, Xiaomao Guo

**Affiliations:** ^1^Cancer Institute, Fudan University Shanghai Cancer Center, Shanghai 200032, China; ^2^Department of Radiation Oncology, Fudan University Shanghai Cancer Center, Shanghai 200032, China; ^3^Department of Oncology, Shanghai Medical College, Fudan University, Shanghai 200032, China; ^4^Department of Laboratory Medicine, Laboratory of Molecular Biology of Huadong Hospital affiliated to Fudan University, Shanghai 200040, China; ^5^Department of Urology, Fudan University Shanghai Cancer Center, Shanghai 200032, China; ^6^Department of Pathology, Fudan University Shanghai Cancer Center, Shanghai 200032, China; ^7^College of Pharmacy, Seoul National University, Seoul, 151-742, Republic of Korea

## Abstract

The neddylation pathway has been recognized as an attractive anticancer target in several malignancies, and its selective inhibitor, MLN4924, has recently advanced to clinical development. However, the anticancer effect of this compound against prostate cancer has not been well investigated. In this study, we demonstrated that the neddylation pathway was functional and targetable in prostate cancer cells. Specific inhibition of this pathway with MLN4924 suppressed the proliferation and clonogenic survival of prostate cancer cells. Mechanistically, MLN4924 treatment inhibited cullin neddylation, inactivated Cullin-RING E3 ligases (CRLs), and led to accumulation of tumor-suppressive CRLs substrates, including cell cycle inhibitors (p21, p27, and WEE1), NF-*κ*B signaling inhibitor I*κ*B*α*, and DNA replication licensing proteins (CDT1 and ORC1). As a result, MLN4924 triggered DNA damage, G2 phase cell cycle arrest, and apoptosis. Taken together, our results demonstrate the effectiveness of targeting the neddylation pathway with MLN4924 in suppressing the growth of prostate cancer cells, implicating a potentially new therapeutic approach for the men's cancer.

## 1. Introduction

Prostate cancer is the most common malignancy among elderly men, representing the second leading cause of male cancer death in developed countries [1, 2]. With the aging population in the coming year, the incidence of prostate cancer will be annually rising. Although prostate cancer patients own favorable 5-year overall survival in general [3], a substantial proportion of patients with an initial response to medical or surgical castration suffers from treatment failure due to acquired hormone resistance [4]. Chemotherapeutic options for prostate cancer patients are historically limited largely because prostate cancer is insensitive to most chemotherapeutics. In order to overcome such limitations, safer and more effective therapeutic agents are needed.

Protein neddylation is a newly characterized protein posttranslational modification in eukaryotic cells by adding NEDD8, an ubiquitin-like molecule, to target proteins [5–8]. Similar to protein ubiquitination, NEDD8 is firstly activated by NEDD8-activating enzyme E1 (also known as NAE, a heterodimer composed of NAE1 and UBA3 subunits), the activated NEDD8 is then transferred to NEDD8-conjugating enzyme E2 (UBC12 or UBE2F), and finally NEDD8-conjugating enzyme E2 collaborates with substrate-specific NEDD8-E3 ligase to covalently conjugate the NEDD8 to its target substrates [9]. The best-characterized NEDD8 substrates are the cullin family proteins that serve as the scaffold components of cullin-RING E3 ligases (CRLs) [10, 11]. CRLs, the largest cellular ubiquitin ligase family, mediate proteasomal degradation of a variety of cellular proteins that function in diverse biological processes whereas their dysfunction leads to carcinogenesis [12–14].

Neddylation-CRLs axis has emerged as a novel anticancer strategy, as evidenced by the antitumor activity of the NEDD8-activating enzyme (NAE) inhibitor MLN4924 [14–16]. This small molecule binds to NAE at the active site and forms a covalent NEDD8-MLN4924 adduct that blocks the subsequent enzymatic cascades for protein neddylation [17, 18]. By doing so, MLN4924 blocks cullin neddylation, inactivates CRLs, and thus leads to accumulation of CRLs substrates and growth suppression of cancer cells [14, 19, 20]. Due to its significant anticancer efficacy and well-tolerated toxicity in preclinical studies, MLN4924 has been advanced into phase I clinical trials for several malignancies [21, 22]. In the present study, we reported that neddylation pathway was activated in prostate cancer cells whereas inhibition of this pathway by MLN4924 exerted significant anticancer efficacy in prostate cancer cells. Our findings lay the foundation for the future development of MLN4924 as a potential treatment of prostate cancer.

## 2. Materials and Methods

### 2.1. Cell Lines and Drug Solutions

Prostate cancer cell lines DU145, LNCap, and PC3 were purchased from American Type Culture Collection and grown in RPMI1640 with 10% fetal bovine serum. Neddylation pathway inhibitor MLN4924, proteasome inhibitors Bortezomib, and MG132 were each dissolved in dimethyl sulfoxide (DMSO) and kept in −20°C before use [23].

### 2.2. Cell Proliferation Assay

Cells were seeded in 96-well plates in triplicate (3000 cells per well) and treated with MLN4924 at various doses. After treatment for 96 hours, cell viability was measured with ATPlite kit (Perkin Elmer), according to the manufacturer's instructions [24].

### 2.3. Clonogenic Cell Survival Assay

Cells were seeded into 6-well plates in triplicate (250 cells per well). Twenty-four hours later, the old culture media were replaced with fresh media in the presence or absence of MLN4924, followed by incubation at 37°C for 12 days. After fixation with 0.2% crystal violet, colonies containing more than 50 individual cells were counted [23].

### 2.4. Immunoblotting (IB)

Cells were harvested and cell lysates were extracted for immunoblotting as described [25], using antibodies against NAE1, UBA3, UBC12, UBE2F, DCN-1, CDT1, ROC1, NEDD8, cullin1, p21, p27, WEE1, phospho-histone H3 (p-H3), total-histone H3 (t-H3), phospho-H2AX (p-H2AX), total H2AX (t-H2AX), total-I*κ*B*α* (t-I*κ*B*α*), phospho-I*κ*B*α* (p-I*κ*B*α*), cleaved Caspase-3 (c-Caspase-3), cleaved PARP (c-PARP), and GAPDH.

### 2.5. Fluorescence Activated Cell Sorting (FACS) Analysis

After treatment with MLN4924, cells were trypsinized, washed with PBS, and fixed in ice-cold 70% ethanol. For cell cycle analysis, cells were stained with propidium iodide and analyzed by CyAn ADP (Beckman Coulter). Data were analyzed with ModFit LT software [23].

### 2.6. Statistical Analysis

Data are presented as mean ± standard error of the mean (SEM). GraphPad Prism5 software was adopted to assess the statistical differences. The unpaired 2-tailed *t* test was performed for the comparison of two groups, and the level of significance was set at *P < 0.05, **P < 0.01, ***P < 0.0001.

## 3. Results

### 3.1. The Neddylation Pathway Was Activated and Targetable in Prostate Cancer Cells

**Figure 1:**
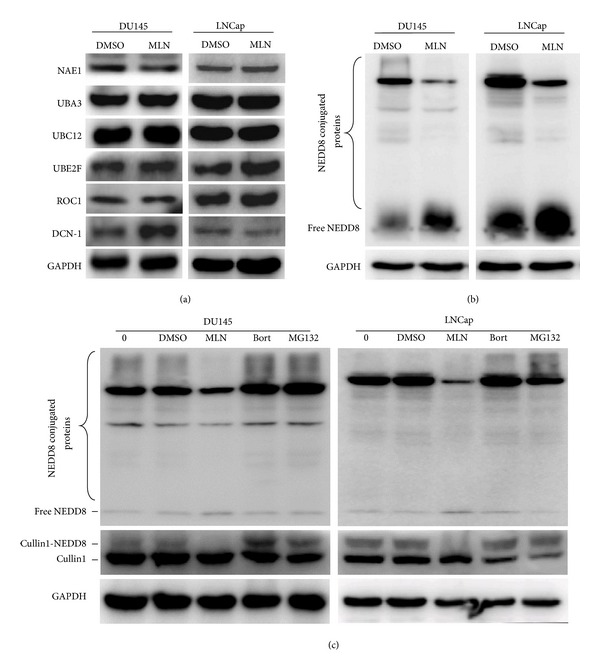
The neddylation pathway was functional and targetable in prostate cancer cells. (a) The components of the neddylation pathway were expressed in prostate cancer cells. Subconfluent cells were subjected to MLN4924 treatment (1 *μ*M) for 12 hours and harvested for immunoblotting (IB) using antibodies against NAE1, UBA3, UBC12, UBE2F, ROC-1, and DCN-1 with GAPDH as a loading control. (b) Neddylation  pathway was activated and targetable in prostate cancer cells. Cells were subjected to MLN4924 (1 *μ*M) for 12 hours and harvested for IB using antibodies against NEDD8 with GAPDH as a loading control. (c) MLN4924 specifically inhibited neddylation pathway. Cells were subjected to MLN4924 (1 *μ*M), Bortezomib (1 *μ*M), or MG132 (20 *μ*M) for 4 hours and harvested for IB using antibodies against NEDD8 and cullin1 with GAPDH as a loading control. MLN, MLN4924; Bort, Bortezomib.

To evaluate the activation status of the neddylation pathway in prostate cancer cells, the expression of key components of the neddylation pathway was examined. As shown in Figure 1(a), NEDD8-activating enzyme E1 (NAE1 and UBA3), NEDD8-conjugating enzyme E2 (UBC12 and UBE2F), and NEDD8-E3 ligases (DCN-1 and ROC1) were expressed in high levels, suggesting the activation of neddylation pathway in prostate cancer cells. In addition, both conjugated and free NEDD8 were revealed to be highly expressed in prostate cancer cells (Figure 1(b)).

The activation of neddylation pathway renders it a potential anticancer target in prostate cancer cells. To test this hypothesis, an effort to suppress neddylation pathway was made by using NAE inhibitor MLN4924. As shown in Figure 1(b), global protein neddylation was obviously suppressed by MLN4924 while free NEDD8 accumulated dramatically in treated cells, demonstrating the functional and targetable status of neddylation pathway in prostate cancer cells. Furthermore, we determined the specificity of MLN4924 for inhibition of the neddylation pathway when compared to Bortezomib (originally codenamed PS-341) and MG132, two classical proteasome inhibitors, and found that MLN4924, but neither Bortezomib nor MG132, specifically inhibited global protein neddylation and cullin neddylation (Figure 1(c)). These results demonstrate that MLN4924 specifically blocks protein neddylation in prostate cancer cells.

### 3.2. MLN4924 Inhibited the Growth of Prostate Cancer Cells

**Figure 2:**
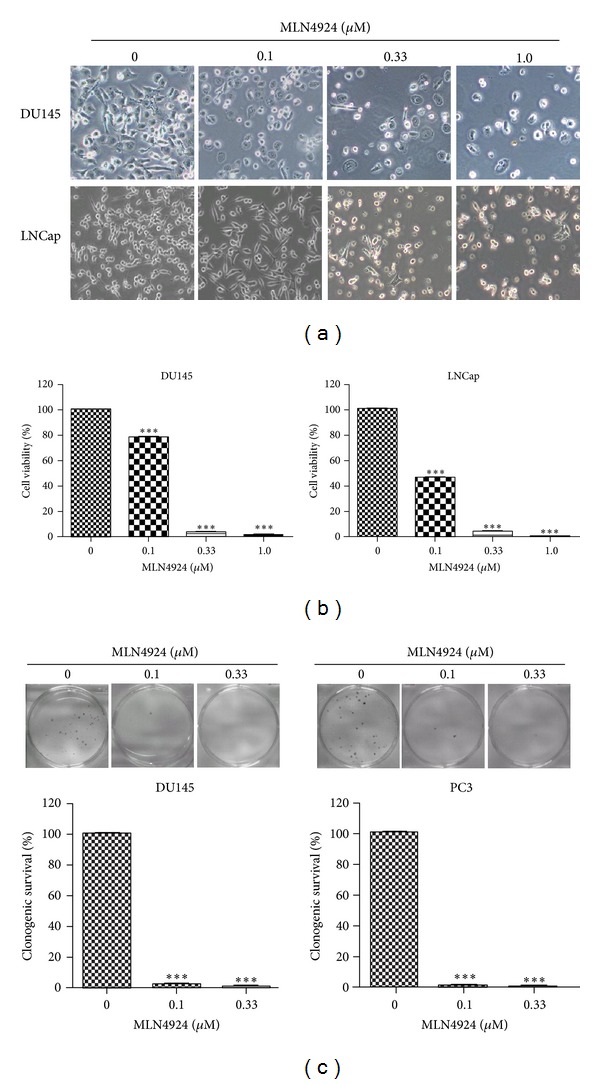
MLN4924 inhibited prostate cancer cell growth. (a) Morphological observations of MLN4924-treated cells. Cells were treated with indicated concentrations of MLN4924 for 48 hours and then photographed. (b) MLN4924 inhibited the proliferation of prostate cancer cells. Cells were seeded in 96-well plates in triplicate and treated with indicated concentrations of MLN4924 for 96 hours; cell viability was determined by ATPlite kit (***P < 0.0001, *n* = 3). (c) MLN4924 inhibited clonogenic cell survival of prostate cancer cells. DU145 and PC3 cells were seeded into 60 mm dishes in duplicate and then grown in the presence or absence of MLN4924 for 12 days. The colonies with more than 50 cells were counted, following crystal violet staining (***P < 0.0001, *n* = 3).

Next we determined the sensitivity of two prostate cancer cell lines to MLN4924. Morphological observations showed that MLN4924 significantly inhibited the proliferation of prostate cancer cells. Moreover, MLN4924-treated cells were shrunk and became round, indicating that the cells were undergoing the apoptosis (Figure 2(a)). Consistently, cell viability assay revealed that MLN4924 induced a dose-dependent impairment of cell viability (Figure 2(b)). In addition, MLN4924 effectively suppressed colony formation in a standard clonogenic survival assay in prostate cancer cells (Figure 2(c)). These data demonstrate that MLN4924 is a potent inhibitor of cell proliferation and survival in prostate cancer cell lines.

### 3.3. MLN4924 Inhibited Cullin Neddylation and Inactivated CRLs

**Figure 3:**
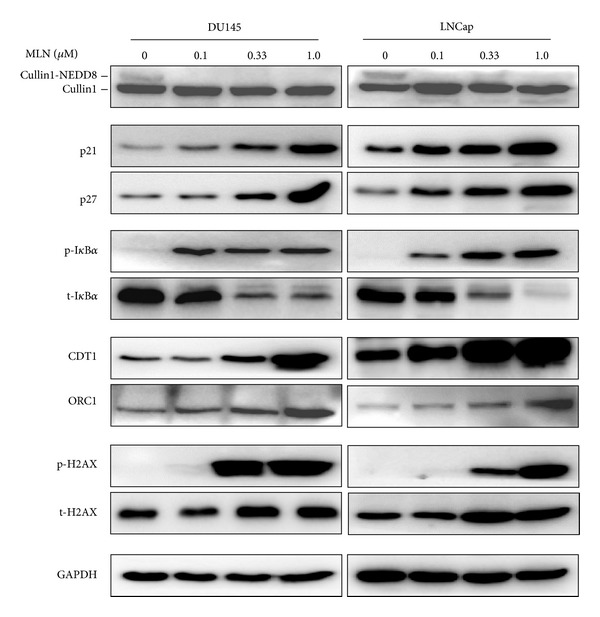
MLN4924 induced accumulation of CRLs substrates and triggered DNA damage in prostate cancer cells. Subconfluent cells were treated with MLN4924 (0, 0.1, 0.33, 1.0 *μ*M) for 48 hours, followed by IB analysis using antibodies against p21, p27, p-I*κ*B*α*, t-I*κ*B*α*, CDT1, ORC1, p-H2AX, and t-H2AX with GAPDH as a loading control.

To address the potential mechanisms underlying the inhibitory effect of MLN4924 on the growth of prostate cancer cells, the expression of a panel of tumor-suppressive CRLs substrates was determined in treated cells. As shown in Figure 3, cullin neddylation was completely blocked by MLN4924, indicating the inactivation of CRLs. As a result, CRLs substrates, including cell cycle inhibitors (p21, p27), NF-*κ*B signaling inhibitor I*κ*B*α*, and DNA replication licensing proteins (CDT1 and ORC1), were accumulated upon MLN4924 treatment. Also, we found that the expression level of phosphorylation H2AX (Figure 3), a classical DNA damage marker, was elevated in MLN4924-treated cells, which was very likely triggered by the accumulation of CDT1 and ORC1 [23].

### 3.4. Inactivation of CRLs by MLN4924 Induced Cell Cycle Arrest and Apoptosis

**Figure 4:**
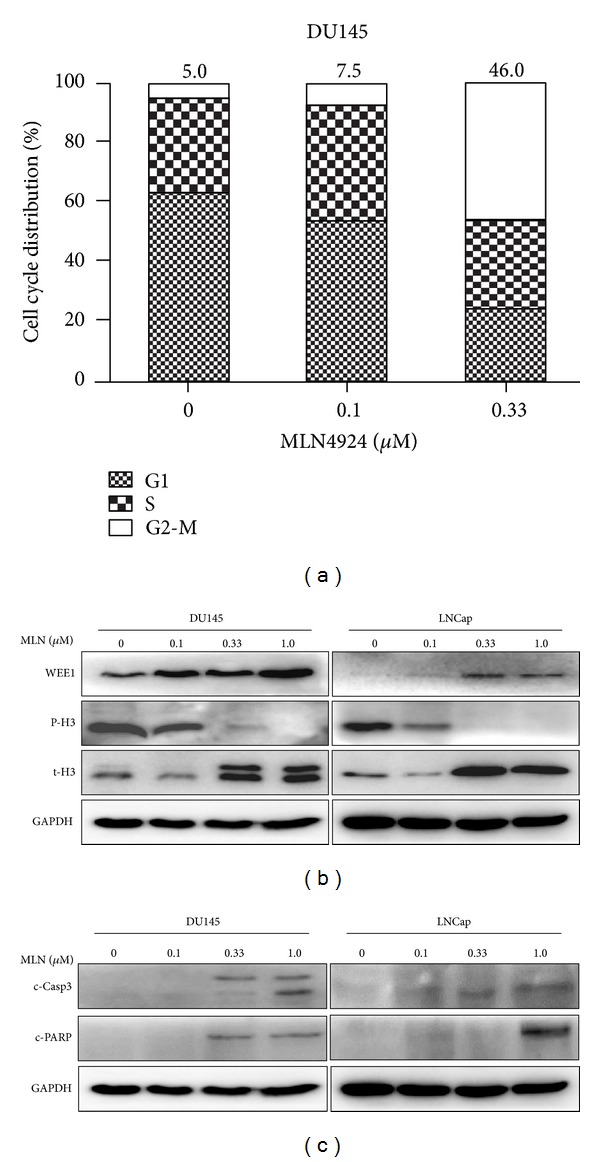
MLN4924 induced G2 phase cell cycle arrest and apoptosis in prostate cancer cells. Subconfluent cells were treated with MLN4924. Forty-eight hours later, one portion of cells was used for cell cycle profile analysis (a), while the other portion was subjected to IB analysis ((b) and (c)). (a) MLN4924 induced G2 phase cell cycle arrest. Cell percentages at G2 phase were 5%, 7.5%, and 46% respectively, when treated with MLN4924 at 0, 0.1 and 0.33 *μ*M. (b) IB analysis to determine the expression of WEE1 and p-histone H3. (c) MLN4924 induced apoptosis in prostate cancer cells. Cells were treated with MLN4924 for 48 hours, followed by IB analysis using antibodies against c-Caspase-3 and c-PARP with GAPDH as a loading control.

To further investigate the nature of MLN4924-mediated growth suppression of prostate cancer cells, we performed the cell cycle profile analysis and found that MLN4924 notably triggered G2/M cell-cycle arrest in DU145 cell lines (Figure 4(a)). To further determine at which phase cells were arrested, we examined the expression of G2-M phase transition inhibitor WEE1 and the mitotic marker p-histone H3. As shown in Figure 4(b), MLN4924 significantly induced the increase of WEE1 and decrease of p-histone H3, revealing that MLN4924-treated cells were arrested at G2 phase. Moreover, MLN4924 treatment induced the expression of cleaved Caspase-3 and cleaved PARP (the biochemical indicators of apoptotic induction) (Figure 4(c)), which was consistent with previous observation (Figure 2(a)) that MLN4924-treated cells displayed apoptosis-like morphology.

## 4. Discussion

With the aging population, the incidence of prostate cancer appears to steadily increase in recent years. However, the current systemic therapy is far from satisfaction due to (1) acquired drug resistance, (2) severe treatment-related adverse effects, and (3) low anticancer efficacy, which ultimately results in recurrence and metastasis. Therefore, new systemic therapy strategies are in urgent need to improve the currently available prostate cancer treatment. The neddylation pathway was recently found to be overactivated in a couple of cancer types [18, 26] and has been considered a promising anticancer target [18] in a number of malignancies. The efforts to screen for small-molecular inhibitor against neddylation pathway have led to the discovery of MLN4924, an inhibitor of NAE. By blocking neddylation of cullin, the best-characterized target of neddylation pathway thus far, MLN4924 has the ability to inactivate CRLs, leads to accumulation of its substrates, and thus eventually leads to suppression of several solid tumors and hematological malignancies both* in vitro* and* in vivo* [9, 21]. In the present study, we found that the neddylation pathway was activated in prostate cancer cells. Moreover, we found that MLN4924 was potent in inhibiting tumor growth in both hormone-sensitive (LNCap) and hormone-resistant (DU145) human prostate carcinoma cell lines.

**Figure 5:**
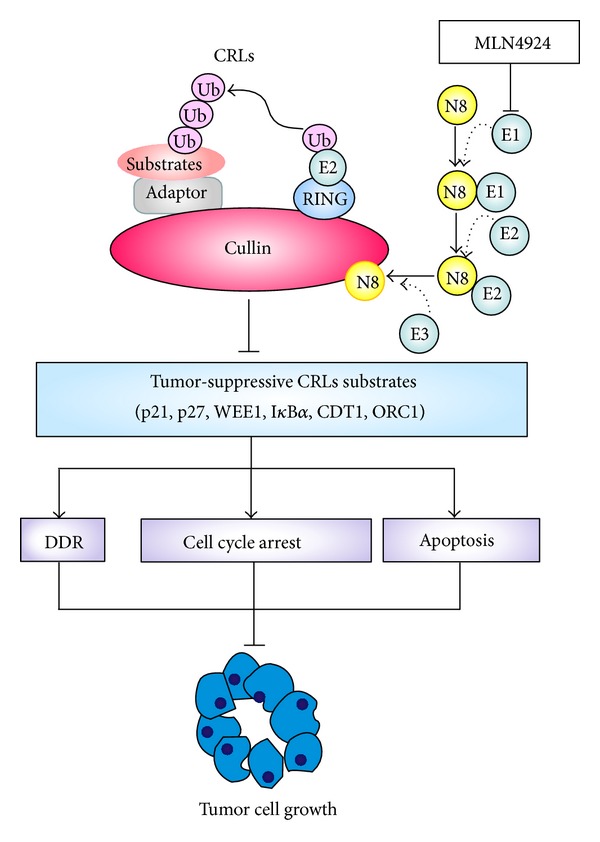
The proposed mechanism underlying the inhibitory effect of MLN4924 in prostate cancer cells. MLN4924 inactivates CRLs by inhibiting cullin neddylation and thus induces accumulation of CRLs substrates. These consequently trigger a series of critical cellular effects including DNA damage response, G2 cycle arrest, and apoptosis, which are responsible for growth suppression of prostate cancer cells. DDR, DNA damage response.

Previous studies reported that blockage of cullin neddylation by MLN4924 was enabled to inactivate CRLs and thus induced multiple cellular effects, including G2 phase arrest, DNA damage response, and apoptosis/senescence [14, 18, 25]. Our results demonstrate that similar mechanisms of growth suppression are shared by prostate cancer upon neddylation inhibition. In prostate cancer cells, neddylation inactivation by MLN4924 blocked cullin neddylation, inhibited CRLs activity, and thus triggered DNA damage, cell cycle arrest, and apoptosis by inducing the accumulation of well-known CRLs substrates, including (1) cell cycle inhibitors p21, p27, and WEE1; (2) NF-*κ*B inhibitor I*κ*B*α*; and (3) DNA replication licensing proteins CDT1 and ORC1 (Figure 5) [14, 23, 27]. These observations suggest that protein neddylation is a conserved signaling pathway essential for the survival of prostate cancer cells. Collectively, targeting neddylation is feasible and reasonable for the treatment of prostate cancer.

## References

[1] Siegel R, Desantis C, Virgo K (2012). Cancer treatment and survivorship statistics, 2012. *CA Cancer Journal for Clinicians*.

[2] Ferlay J, Autier P, Boniol M, Heanue M, Colombet M, Boyle P (2007). Estimates of the cancer incidence and mortality in Europe in 2006. *Annals of Oncology*.

[3] Zhou Z, Zhu X, Xia J (2013). Short-term versus long-term hormone therapy plus radiotherapy or prostatectomy for prostate cancer: A systematic review and meta-analysis. *Journal of Cancer Research and Clinical Oncology*.

[4] Feldman BJ, Feldman D (2001). The development of androgen-independent prostate cancer. *Nature Reviews Cancer*.

[5] Xirodimas DP (2008). Novel substrates and functions for the ubiquitin-like molecule NEDD8. *Biochemical Society Transactions*.

[6] Xirodimas DP, Saville MK, Bourdon JC, Hay RT, Lane DP (2004). Mdm2-mediated NEDD8 conjugation of p53 inhibits its transcriptional activity. *Cell*.

[7] Zuo W, Huang F, Chiang YJ (2013). c-Cbl-mediated neddylation antagonizes ubiquitination and degradation of the TGF-*β* type II receptor. *Molecular Cell*.

[8] Loftus SJ, Liu G, Carr SM, Munro S, la Thangue NB (2012). NEDDylation regulates E2F-1-dependent transcription. *EMBO Reports*.

[9] Soucy TA, Smith PG, Rolfe M (2009). Targeting NEDD8-activated cullin-RING ligases for the treatment of cancer. *Clinical Cancer Research*.

[10] Sakata E, Yamaguchi Y, Miyauchi Y (2007). Direct interactions between NEDD8 and ubiquitin E2 conjugating enzymes upregulate cullin-based E3 ligase activity. *Nature Structural & Molecular Biology*.

[11] Saha A, Deshaies RJ (2008). Multimodal activation of the ubiquitin ligase SCF by Nedd8 conjugation. *Molecular Cell*.

[12] Jia L, Sun Y (2011). SCF E3 ubiquitin ligases as anticancer targets. *Current Cancer Drug Targets*.

[13] Teixeira LK, Reed SI (2013). Ubiquitin ligases and cell cycle control. *Annual Review of Biochemistry*.

[14] Soucy TA, Smith PG, Milhollen MA (2009). An inhibitor of NEDD8-activating enzyme as a new approach to treat cancer. *Nature*.

[15] Jia L, Li H, Sun Y (2011). Induction of p21-dependent senescence by an NAE inhibitor, MLN4924, as a mechanism of growth suppression. *Neoplasia*.

[16] Blank JL, Liu XJ, Cosmopoulos K (2013). Novel DNA damage checkpoints mediating cell death induced by the NEDD8-activating enzyme inhibitor MLN4924. *Cancer Research*.

[17] Brownell JE, Sintchak MD, Gavin JM (2010). Substrate-assisted inhibition of ubiquitin-like protein-activating enzymes: the NEDD8 E1 inhibitor MLN4924 forms a NEDD8-AMP mimetic in situ. *Molecular Cell*.

[18] Zhao Y, Xiong X, Jia L, Sun Y (2012). Targeting Cullin-RING ligases by MLN4924 induces autophagy via modulating the HIF1-REDD1-TSC1-mTORC1-DEPTOR axis. *Cell Death & Disease*.

[19] Lin JJ, Milhollen MA, Smith PG, Narayanan U, Dutta A (2010). NEDD8-targeting drug MLN4924 elicits DNA rereplication by stabilizing Cdt1 in S phase, triggering checkpoint activation, apoptosis, and senescence in cancer cells. *Cancer Research*.

[20] Mackintosh C, García-Domínguez DJ, Ordóñez JL (2013). WEE1 accumulation and deregulation of S-phase proteins mediate MLN4924 potent inhibitory effect on Ewing sarcoma cells. *Oncogene*.

[21] Soucy TA, Dick LR, Smith PG, Milhollen MA, Brownell JE (2010). The NEDD8 conjugation pathway and its relevance in cancer biology and therapy. *Genes & Cancer*.

[22] Tanaka T, Nakatani T, Kamitani T (2013). Negative regulation of NEDD8 conjugation pathway by novel molecules and agents for anticancer therapy. *Current Pharmaceutical Design*.

[23] Luo Z, Yu G, Lee HW (2012). The Nedd8-activating enzyme inhibitor MLN4924 induces autophagy and apoptosis to suppress liver cancer cell growth. *Cancer Research*.

[24] Jia L, Soengas MS, Sun Y (2009). ROC1/RBX1 E3 ubiquitin ligase silencing suppresses tumor cell growth via sequential induction of G2-M arrest, apoptosis, and senescence. *Cancer Research*.

[25] Li L, Liu B, Dong T (2013). Neddylation pathway regulates the proliferation and survival of macrophages. *Biochemical and Biophysical Research Communications*.

[26] Salon C, Brambilla E, Brambilla C, Lantuejoul S, Gazzeri S, Eymin B (2007). Altered pattern of Cul-1 protein expression and neddylation in human lung tumours: relationships with CANDI and cyclin E protein levels. *Journal of Pathology*.

[27] Jia L, Bickel JS, Wu J (2011). RBX1 (RING box protein 1) E3 ubiquitin ligase is required for genomic integrity by modulating DNA replication licensing proteins. *Journal of Biological Chemistry*.

